# APOE effects on regional tau in preclinical Alzheimer’s disease

**DOI:** 10.1186/s13024-022-00590-4

**Published:** 2023-01-04

**Authors:** Christina B. Young, Emily Johns, Gabriel Kennedy, Michael E. Belloy, Philip S. Insel, Michael D. Greicius, Reisa A. Sperling, Keith A. Johnson, Kathleen L. Poston, Elizabeth C. Mormino, for the Alzheimer’s Disease Neuroimaging Initiative, the A4 Study Team

**Affiliations:** 1 Stanford University School of Medicine, 453 Quarry Rd., Palo Alto, Stanford, CA 94304 USA; 2 University of California San Francisco, San Francisco, CA USA; 3 Brigham and Women’s Hospital, Boston, MA USA; 4 Massachusetts General Hospital, Boston, MA USA

**Keywords:** *APOE*, e2, e4, Amyloid, Tau, PET, Preclinical Alzheimer’s disease, Clinically unimpaired

## Abstract

**Background:**

*APOE* variants are strongly associated with abnormal amyloid aggregation and additional direct effects of *APOE* on tau aggregation are reported in animal and human cell models. The degree to which these effects are present in humans when individuals are clinically unimpaired (CU) but have abnormal amyloid (Aβ+) remains unclear.

**Methods:**

We analyzed data from CU individuals in the Anti-Amyloid Treatment in Asymptomatic AD (A4) and Longitudinal Evaluation of Amyloid Risk and Neurodegeneration (LEARN) studies. Amyloid PET data were available for 4486 participants (3163 Aβ-, 1323 Aβ+) and tau PET data were available for a subset of 447 participants (55 Aβ-, 392 Aβ+). Linear models examined *APOE* (number of e2 and e4 alleles) associations with global amyloid and regional tau burden in medial temporal lobe (entorhinal, amygdala) and early neocortical regions (inferior temporal, inferior parietal, precuneus). Consistency of *APOE*4 effects on regional tau were examined in 220 Aβ + CU and mild cognitive impairment (MCI) participants from the Alzheimer’s Disease Neuroimaging Initiative (ADNI).

**Results:**

*APOE*2 and *APOE*4 were associated with lower and higher amyloid positivity rates, respectively. Among Aβ+ CU, e2 and e4 were associated with reduced (−12 centiloids per allele) and greater (+15 centiloids per allele) continuous amyloid burden, respectively. *APOE*2 was associated with reduced regional tau in all regions (-0.05 to -0.09 SUVR per allele), whereas *APOE*4 was associated with greater regional tau (+0.02 to +0.07 SUVR per allele). *APOE* differences were confirmed by contrasting e3/e3 with e2/e3 and e3/e4. Mediation analyses among Aβ+ s showed that direct effects of e2 on regional tau were present in medial temporal lobe and early neocortical regions, beyond an indirect pathway mediated by continuous amyloid burden. For e4, direct effects on regional tau were only significant in medial temporal lobe. The magnitude of protective e2 effects on regional tau was consistent across brain regions, whereas detrimental e4 effects were greatest in medial temporal lobe. *APOE*4 patterns were confirmed in Aβ+ ADNI participants.

**Conclusions:**

*APOE* influences early regional tau PET burden, above and beyond effects related to cross-sectional amyloid PET burden. Therapeutic strategies targeting underlying mechanisms related to *APOE* may modify tau accumulation among Aβ+ individuals.

**Supplementary Information:**

The online version contains supplementary material available at 10.1186/s13024-022-00590-4.

## Background

*APOE*4 is the strongest genetic predictor of sporadic Alzheimer’s disease (AD) dementia [1–4] and is consistently associated with risk of abnormal amyloid during the stages preceding dementia onset [5–7]. *APOE* influences amyloid accumulation through interactions with production, fibrillization, and clearance mechanisms, as well as interactions between *APOE*, amyloid, tau, neuroinflammation, and neuronal structure and function [8–10]. On average, *APOE*4 carriers typically reach the threshold for abnormal amyloid (Aβ+) approximately 10–15 years before e3/3 carriers, whereas amyloid positivity is delayed by approximately 4 years in e2 carriers compared to e3/e3 carriers [11].

Tau abnormalities are a stronger predictor of concurrent cognitive ability and future cognitive impairment than Aβ status alone [12–14]. Although both amyloid and tau abnormalities are strongly related such that elevated tau is very uncommon among Aβ- individuals, there is substantial variability in the magnitude of tau PET uptake within Aβ+ individuals [12, 15–20], highlighting that additional factors beyond amyloid contribute to downstream tau accumulation. Interestingly, there is growing evidence suggesting that *APOE* may directly impact variability in tau accumulation. For instance, tau PET studies in Aβ+ patients across the AD clinical spectrum have shown a more medial temporal lobe (MTL)-dominant pattern of tau pathology in e4 carriers compared to non-carriers [16, 21] even after adjusting for global amyloid burden [22, 23]. This suggests that *APOE* genotype may influence disease progression even after amyloid positivity is reached. This may also partially explain why in early clinical trials for AD dementia patients, which did not yet routinely assess amyloid status, e4 carriers declined more rapidly than non-e4 carriers [24].

However, analyses examining *APOE* effects on tau PET specifically among clinically unimpaired (CU) participants have found mixed results. For instance, Lowe and colleagues [25] reported no effect of e4 on tau, and Ramanan and colleagues [26] found similar null effects after controlling for global levels of amyloid. In contrast, Ossenkoppele and colleagues [27] found a significant effect of e4 on entorhinal tau and Ghisays and colleagues [28] reported that e4 modified the association between age and entorhinal tau. Thus, the extent to which *APOE*4 influences early regional tau burden in Aβ+ CU remains unclear [29]. Additionally, little has been established regarding whether *APOE*2 is associated with reduced tau burden in Aβ+ individuals. A primary barrier to examining e2 effects is that  e2 carriership is uncommon in Aβ+ CU individuals. Within Aβ+ CU individuals, approximately 4–8% will be e2 carriers whereas around 50% will be e4 carriers [29–31]. One study examining CU combined with mild cognitive impairment (MCI) showed comparable levels of regional tau burden and tau accumulation over time in 45 e2 carriers compared to 257 e3/e3 carriers [32], though only 15 of the 45 e2 carriers were Aβ+. Post-mortem work examining the full disease spectrum from CU to AD dementia has shown mixed results, with one study of 411 e2 carriers showing no effect on Braak staging after controlling for neuritic amyloid plaque severity [33], and another study of 163 e2 carriers showing fewer tau tangles in the Aβ+ e2 group compared to Aβ+ e2 non-carriers [34]. To date, it is likely that studies focused on CU cohorts have been underpowered to determine whether e2 effects influence tau accumulation among Aβ+ .

The overall goal of the present study was to establish whether *APOE* genotype influences early tau burden in preclinical AD beyond effects attributable to amyloid burden. We leveraged the Anti-Amyloid Treatment in Asymptomatic AD (A4) and Longitudinal Evaluation of Amyloid Risk and Neurodegeneration (LEARN) amyloid PET screening dataset of 4486 CU [30, 35] along with the tau PET substudy [29, 30] that included 392 Aβ+ CU individuals with tau PET. *APOE*4 effects were additionally confirmed in 220 Aβ+ CU and MCI participants from the Alzheimer’s Disease Neuroimaging Initiative (ADNI). We hypothesized that *APOE* genotype would influence regional tau PET values in the MTL and that these effects would be present after controlling for continuous amyloid burden.

## Methods

The A4 Study is a secondary prevention trial that focused on participants with preclinical AD (i.e., Aβ+ CU) [30]. The LEARN study is a companion to the A4 study focused on Aβ- individuals. After initial telephone and in-clinic screenings to determine study eligibility including cognitive status, 4486 participants underwent amyloid PET scanning to allow for identification of Aβ+ individuals prior to treatment randomization. A subset enriched for  amyloid positivity also underwent tau PET scanning prior to treatment randomization. All A4/LEARN participants completed written informed consent before participation. The ADNI is a public-private partnership launched in 2003 with the primary goal of testing whether serial neuroimaging and biological markers, and clinical and neuropsychological assessments can be combined to measure the progression of MCI and early AD. All ADNI participants provided written informed consent in compliance with local IRBs. For up-to-date information, see www.adni-info.org.

In the A4/LEARN dataset, all participants included in this study (Table 1 and Table S1) were 65–85 years old, CU (Clinical Dementia Rating score = 0, Mini-Mental State Examination score = 25–30, and Logical Memory Delayed Recall score = 6–18), and had completed amyloid PET scans ([18F]-florbetapir). A subset of these participants also completed tau PET scans ([18F]-flortaucipir). Amyloid status was determined using a hybrid quantitative and qualitative method established by the A4/LEARN study team [30]. Global standardized uptake value ratios (SUVRs) for amyloid PET (reference region: whole cerebellum) and regional SUVRs for tau PET (reference region: cerebellar gray) were extracted using previously published pipelines [29]. Briefly, amyloid and tau PET data were processed with in-house scripts using FSL, SPM, and FreeSurfer. Five-minute amyloid and tau PET frames corresponding to 50–70 min and 90–110 min post-injection, respectively, were realigned and summed. Each participant’s MRI data (including aparc+aseg FreeSurfer labels) were coregistered to the summed PET data. Mean uptake values for each aparc+aseg FreeSurfer region were extracted. Cortical amyloid SUVR was calculated following ADNI procedures (see “Florbetapir (AV45) processing methods” from http://adni.loni.usc.edu) and regional tau analyses focused on entorhinal, amygdala, inferior temporal, inferior parietal, and precuneus regions as defined by FreeSurfer.

**Table 1:** Demographic information for the A4/LEARN dataset

	Full Dataset		Tau Subset	
	Aβ- (*n* = 3163)	Aβ+ (*n* = 1323)	Aβ- (*n* = 55)	Aβ+ (*n* = 392)
Age, mean (SD)	71.0 (4.5)	72.1 (4.9)	69.7 (4.3)	72.1 (4.8)
Sex, *n* (%)				
Male	1278 (40%)	545 (41%)	23 (42%)	167 (43%)
Female	1885 (60%)	778 (59%)	32 (58%)	225 (57%)
*APOE* genotype, *n* (%)				
e2/e2	23 (1%)	2 (0%)	0 (0%)	1 (0%)
e2/e3	380 (12%)	69 (5%)	4 (7%)	17 (4%)
e2/e4	74 (2%)	42 (3%)	1 (2%)	13 (3%)
e3/e3	1936 (61%)	481 (36%)	37 (67%)	147 (38%)
e3/e4	684 (22%)	611 (46%)	13 (24%)	182 (46%)
e4/e4	34 (1%)	105 (8%)	0 (0%)	25 (6%)
Missing	32 (1%)	13 (1%)	0 (0%)	7 (2%)
Amyloid Centiloid, mean (SD)	–	–	4.0 (9.9)	54.8 (30.3)

In the ADNI dataset, only participants with a tau PET scan ([18F]-flortaucipir), an amyloid positive PET scan ([18F]-florbetaben (FBB) or [18F]-florbetapir (FBP)), available *APOE* status, and a diagnosis of CU or MCI within 1 year of the tau PET scan were included (Table 2). Amyloid PET SUVRs and cutoffs for amyloid status (reference region: whole cerebellum) as well as regional tau PET (reference region: inferior cerebellum) were downloaded from the ADNI LONI website.

**Table 2:** Demographic information for the ADNI dataset

	FBP (Aβ+ *n* = 109)	FBB (Aβ+ *n* = 111)	Overall (Aβ+ *n* = 220)
Age, mean (SD)	78.8 (7.3)	73.1 (7.2)	75.9 (7.8)
Sex, *n* (%)			
Male	54 (50%)	55 (50%)	109 (50%)
Female	55 (50%)	56 (50%)	111 (50%)
Diagnosis, *n* (%)			
CU	45 (41%)	49 (44%)	94 (43%)
MCI due to AD	57 (52%)	59 (53%)	116 (53%)
MCI due to Other	7 (6%)	3 (3%)	10 (5%)
*APOE* genotype, *n* (%)			
e2/e2	0 (0%)	0 (0%)	0 (0%)
e2/e3	8 (7%)	1 (1%)	9 (4%)
e2/e4	3 (3%)	1 (1%)	4 (2%)
e3/e3	39 (36%)	35 (32%)	74 (34%)
e3/e4	46 (42%)	58 (52%)	104 (47%)
e4/e4	13 (12%)	16 (14%)	29 (13%)
Amyloid Centiloid, mean (SD)	59.4 (30.5)	69.2 (35.3)	64.3 (33.3)
Years between tau and amyloid PET scans, mean (SD)	−2.3 (1.9)	0.0 (0.2)	−1.2 (1.8)

For both A4/LEARN and ADNI amyloid data, amyloid SUVRs were converted to the common Centiloid (CL) scale using previously published equations [36] (Fig. S1). This conversion allows for direct comparison of data collected from different tracers and from different studies. ADNI analyses combined [18F]-florbetaben and [18F]-florbetapir ligands using CL values.

### Statistical analysis

Data were analyzed using R v4.1.2. Analyses primarily focused on the A4/LEARN dataset given the substantially larger sample of Aβ+ CU individuals with tau PET. First, to examine the association between *APOE* and amyloid burden, logistic regression models using the questionr package to obtain odds ratios examined the effects of e2 and e4 dosage on dichotomous amyloid status (e.g., e3/e3 carriers were coded with an e2 dosage of 0 and an e4 dosage of 0; e3/e4 carriers were coded with an e2 dosage of 0 and an e4 dosage of 1; e2/e4 carriers were coded with an e2 dosage of 1 and an e4 dosage of 1). Within Aβ+s, linear regression models also examined the effects of e2 and e4 dosage on continuous amyloid burden. Models were repeated to include age*APOE interactions and sex*APOE interactions. Second, across Aβ-s and Aβ+s, linear regression models examined the effects of continuous amyloid on regional tau. Third, linear regression models examined the effects of e2 and e4 dosage on regional tau within Aβ+s only. Primary analyses included all genotypes. Additionally, we conducted a series of sensitivity analyses to ensure that the APOE effects on regional tau were not primarily driven by the presence of uncommon genotypes or our allele dosage modeling approach: (1) linear regression models with e2 and e4 dosage were repeated after excluding e2/e2 and e4/e4 homozygotes, (2) linear regression models with e2 and e4 dosage were repeated after excluding e2/e4 carriers, (3) instead of modeling e2 and e4 dosage, linear regression models contrasted common *APOE* genotype groups (e2/e3 vs. e3/e3, e3/e4 vs. e3/e3). Finally, mediation models using the lavaan package examined whether continuous amyloid mediated the association between *APOE* dosage and regional tau in Aβ+s. Bootstrapping procedures (*n* = 10,000) were used to determine significance of effects in mediation models as well as to compare e2 and e4 effect sizes. All models controlled for mean centered age (71.3 years) and sex.

Analyses were repeated in Aβ+ CU and MCI participants from ADNI. Given the small number of Aβ+ e2 individuals in ADNI (*n* = 7 CU and *n* = 6 MCI), ADNI was only used to examine effects of e4. Mediation models examining whether continuous amyloid mediated the association between e4 dosage and regional tau were repeated in 220 Aβ+ CU and MCI participants. These models also controlled for mean centered age (75.9 years) and sex.

## Results

### APOE predicts amyloid status

**Fig. 1:**
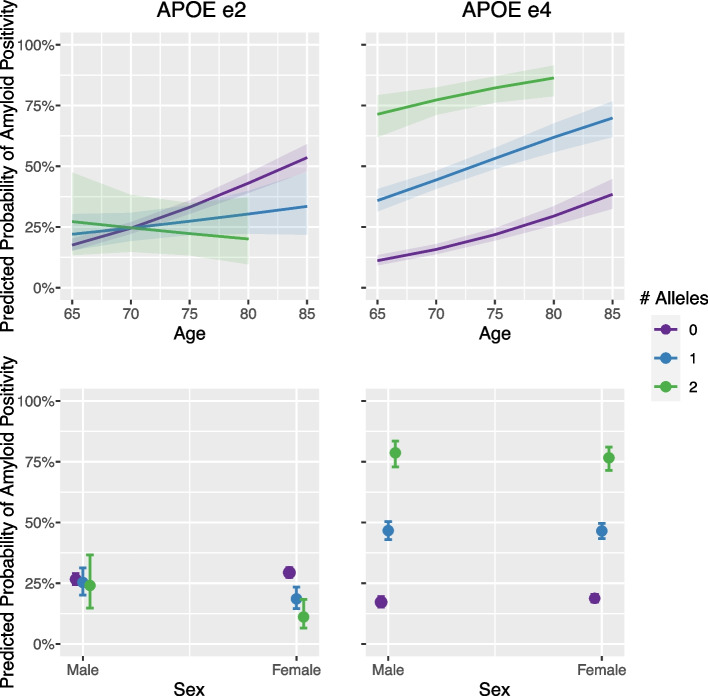
*APOE* interactions with age and sex. The top row shows associations between age and probability of Aβ+ status as a function of *APOE* carriership. The top left panel depicts reduced effects of age on Aβ+ status in e2 carriers. The top right panel shows increasing associations between age and Aβ+ status across all levels of e4 carriership. The bottom row shows associations between sex and probability of Aβ+ status as a function of *APOE* carriership. The bottom left panel depicts protective effects of e2 in females. The bottom right panel shows no significant difference between sexes in probability of Aβ+ status across all levels of e4 carriership

*APOE* genotype was significantly related to amyloid status after accounting for age and sex. As expected, e2 dosage was associated with amyloid negativity and e4 dosage was associated with amyloid positivity (Table 3A). The impact of age on amyloid positivity was reduced in e2 carriers and the protective effects of e2 on amyloid positivity was not observed in males (Table 3A and Fig. 1).

**Table 3:** *APOE* associations with **(A)** amyloid status and **(B)** continuous amyloid burden (centiloids) among Aβ+ clinically unimpaired (CU) individuals. Each column represents a separate regression model. Odds ratios (OR) and their 95% confidence intervals (CI) are provided in (A) and unstandardized betas and their standard errors (SE) are listed in (B)

	A. Predicting amyloid status in 3163 Aβ- and 1323 Aβ+ CUs		B. Predicting continuous amyloid burden in 392 Aβ+ CUs	
	OR (95% CI), *p*-value	OR (95% CI), *p*-value	B (SE), *p*-value	B (SE), *p*-value
*APOE*2	**0.658 (0.526–0.817), *p*** **< 0.001**	0.935 (0.674–1.279), *p* = 0.679	**−11.815 (4.993), *p*** **= 0.019**	**−17.310 (7.499), *p*** **= 0.022**
*APOE*4	**3.909 (3.445–4.444), *p*** **< 0.001**	**4.213 (3.459–5.151), *p*** **< 0.001**	**15.321 (2.401), *p*** **< 0.001**	**16.411 (3.948), *p*** **< 0.001**
Age (Years)	**1.080 (1.064–1.097), *p*** **< 0.001**	**1.092 (1.071–1.114), *p*** **< 0.001**	**1.817 (0.302), *p*** **< 0.001**	**1.456 (0.434), *p*** **< 0.001**
Sex (F vs. M)	1.066 (0.925–1.229), *p* = 0.379	1.198 (0.981–1.466), *p* = 0.078	2.638 (2.922), *p* = 0.367	3.317 (4.354), *p* = 0.447
*APOE*2 * Age	–	**0.946 (0.905–0.989), *p*** **= 0.014**	–	0.565 (1.021), *p* = 0.580
*APOE*4 * Age	–	0.991 (0.964–1.019), *p* = 0.515	–	0.534 (0.526), *p* = 0.310
*APOE*2 * Sex (F vs. M)	–	**0.586 (0.377–0.911), *p*** **= 0.018**	–	9.448 (10.325), *p* = 0.361
*APOE*4 * Sex (F vs. M)	–	0.894 (0.692–1.153), *p* = 0.389	–	−2.021 (4.983), *p* = 0.685

### Global amyloid burden associations with regional tau

**Fig. 2:**
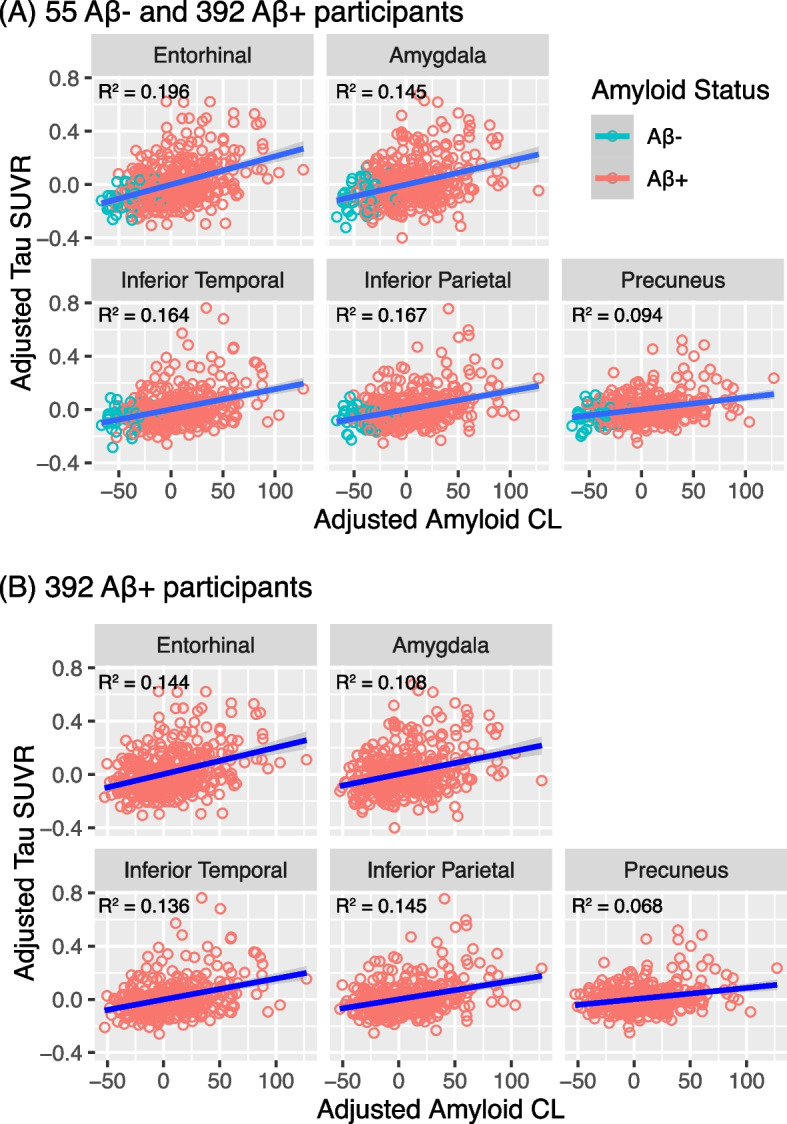
Association between amyloid and tau burden in (**A**) Aβ- and Aβ+ participants and in (**B**) only Aβ+ participants. Plotted tau SUVRs and amyloid centiloids (CLs) are residualized by age and sex

Among Aβ+s with tau PET data, *APOE* genotype was significantly associated with continuous amyloid burden but interactions with age and sex were not significant (Table 3B). Higher continuous amyloid burden was associated with greater regional tau levels across all MTL and early neocortical tau regions (Fig. 2 and Table S2). In general, Aβ- CU individuals showed little evidence of tau elevations. Although the Aβ+ group showed elevated regional tau SUVRs, a wide range was present, with some Aβ+ individuals showing evidence of tau elevations and others showing levels comparable to the Aβ- group (Fig. 2). Overall, among the Aβ+ group, age, sex, and continuous amyloid burden explained 7–15% of the total variance of regional tau (Table S2).

### APOE associations with regional tau

Given the minimal levels of tau in Aβ-s combined with the small sample size of this group (*n* = 55), we next examined the influence of *APOE* on regional tau in Aβ+s only (*n* = 392). *APOE* e2 and e4 were significantly associated with reduced and increased tau, respectively, in MTL and early neocortical regions (Table 4). *APOE*2 effects on reduced tau in amygdala and precuneus were strongest at older ages (Table S3). Results were similar after excluding the single e2/e2 Aβ+ participant and the 25 e4/e4 Aβ+ homozygotes (Table S4) as well as after excluding 13 e2/e4 Aβ+ carriers (Table S5).

**Table 4:** *APOE* associations with regional tau SUVRs in 392 Aβ+s after controlling for age and sex. Each column represents a separate regression model. Unstandardized betas (SE) and p-values are listed

	Entorhinal	Amygdala	Inferior Temporal	Inferior Parietal	Precuneus
*APOE*2	**−0.088 (0.028), *p*** **= 0.002**	**−0.084 (0.028), *p*** **= 0.003**	**−0.055 (0.023), *p*** **= 0.017**	**−0.045 (0.022), *p*** **= 0.037**	**−0.056 (0.017), *p*** **= 0.001**
*APOE*4	**0.056 (0.013), *p*** **< 0.001**	**0.073 (0.013), *p*** **< 0.001**	**0.034 (0.011), *p*** **= 0.002**	**0.032 (0.010), *p*** **= 0.002**	**0.019 (0.008), *p*** **= 0.023**
Age	**0.004 (0.002), *p*** **= 0.034**	**0.006 (0.002), *p*** **= 0.001**	**0.003 (0.001), *p*** **= 0.047**	−0.001 (0.001), *p* = 0.318	0.000 (0.001), *p* = 0.682
Sex (F vs. M)	0.032 (0.016), *p* = 0.052	0.000 (0.016), *p* = 0.998	0.017 (0.013), *p* = 0.211	**0.042 (0.013), *p*** **= 0.001**	0.006 (0.010), *p* = 0.564

**Fig. 3:**
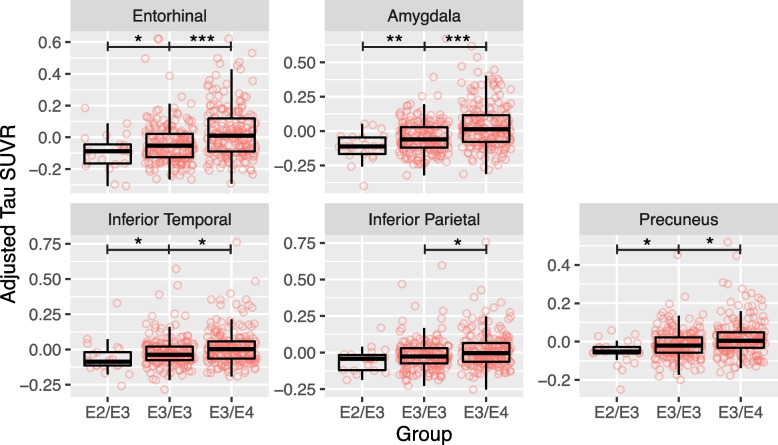
Regional tau differences between Aβ+ *APOE* e2/e3, e3/e3, and e3/e4 groups. Plotted tau SUVRs are residualized by age and sex. Note: * *p* < 0.05, **p < 0.01, ***p < 0.001

*APOE* differences among Aβ+ s were further confirmed by directly contrasting e2/3 (*n* = 17) and e3/4 (*n* = 182) against the e3/3 group (*n* = 147). *APOE* e2/e3 individuals had significantly lower tau SUVRs than e3/e3 individuals in entorhinal cortex, amygdala, inferior temporal, and precuneus (0.05–0.09 SUVRs), whereas e3/e4 individuals had significantly higher tau SUVRs than e3/e3 individuals in all examined MTL and early neocortical regions (0.03–0.08 SUVRs; Fig. 3 and Table S6).

### Direct effects of APOE on regional tau in Aβ+ CU

**Fig. 4:**
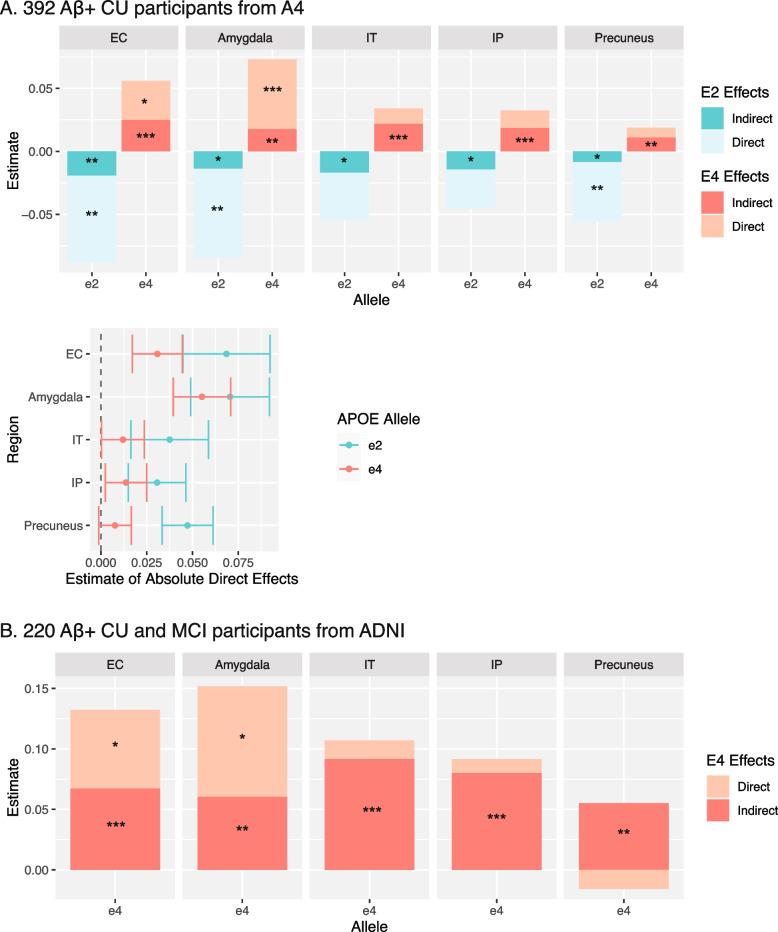
Mediating effects of amyloid (centiloids) on *APOE* and regional tau SUVRs. (**A)** Stacked bar plots depict the total effect, as well as indirect and direct subcomponents, extracted from mediation models examining the effects of *APOE* on regional tau among Aβ+ clinically unimpaired (CU) individuals from A4. A comparison of e2 and e4 direct effect sizes are also shown. Error bars reflect standard error. (**B)** Stacked bar plots depict the total effect, as well as indirect and direct subcomponents, extracted from mediation models examining the effects of *APOE* e4 on regional tau among Aβ+ CU and mild cognitive impairment (MCI) individuals from ADNI. Note: * *p* < 0.05, **p < 0.01, ***p < 0.001

Because *APOE* genotype remains a significant predictor variable of continuous amyloid burden even within Aβ+ s (Fig. 2 and Table S2), we conducted a series of mediation models to determine whether global amyloid burden mediates the association between *APOE* and regional tau in Aβ+ CU individuals (Fig. 4A; Fig. S2A). Continuous amyloid burden accounted for 14–27% of the effect of e2 on regional tau and 21–68% of the effect of e4 on regional tau. For e2, partial mediation was found in both MTL and early neocortical regions with significant direct effects remaining between e2 and regional tau across all regions. In contrast, partial mediation effects were found for e4 in MTL regions only; direct effects of e4 on tau in early neocortical regions were not significant.

The e2 and e4 effect sizes were compared within and across tau regions using bootstrapping procedures (Fig. 4A). The direct effect of e4 on regional tau SUVRs was significantly stronger in the amygdala in comparison to neocortical regions. The strength of direct effects of e2 in MTL were qualitatively larger but not significantly different than those observed in neocortical regions, with the exception of stronger e2 effects in amygdala than inferior parietal tau. Within each tau region, e2 direct effects were qualitatively stronger than e4 direct effects, though these differences were not significant except in precuneus.

### Amyloid mediates the association between APOE4 and tau in Aβ+ CU and MCI

Mediation analyses examining effects of e4 were conducted in 220 Aβ+ CU and MCI individuals from ADNI (Fig. 4B). *APOE*2 effects were not examined given the small sample size of only 13 Aβ+ e2 carriers (9 e2/e3 and 4 e2/e4) in this dataset. This analysis showed that e4 effects on regional tau were 2–3 times larger than e4 effects from the A4/LEARN analyses, presumably because the ADNI cohort included Aβ+ MCI and therefore a broader range of regional tau values. Among Aβ+ CU and MCI, continuous amyloid burden accounted for 40–51% of the variance of the effect of e4 on MTL tau and both indirect and direct effects were significant. In neocortical regions, continuous amyloid burden fully accounted for the variance of the effect of e4, and only indirect pathways through continuous amyloid were significant (i.e., no significant direct effect of e4 on neocortical tau remained). Thus, e4 effects on regional tau were consistent across A4/LEARN and ADNI analyses, with both cohorts showing significant direct effects between e4 and MTL tau PET, and no significant direct effects of e4 on neocortical regions after accounting for continuous amyloid PET burden. All effects were similar when only including the 162 participants who had tau PET and amyloid PET scans within 2 years of each other (Fig. S3).

## Discussion

In a large cohort of Aβ+ CU from the A4/LEARN study, we found that *APOE* genotype is associated with both global amyloid PET burden as well as regional tau PET in MTL and early neocortical regions. Cross-sectional examinations of *APOE*, amyloid, and tau demonstrated both amyloid-mediated and direct effects of e2 and e4 on tau within the MTL. For tau in early neocortical regions, both amyloid-mediated and direct effects were present for e2 whereas only amyloid-mediated effects were present for e4. Our findings provide evidence that *APOE* genotype influences early tau burden in preclinical AD beyond effects that are attributable to amyloid burden as measured with PET. This work highlights that although amyloid positivity is a key driver of downstream tau accumulation, *APOE* genotype additionally influences disease progression through mechanisms directly related to tau accumulation.

*APOE*4 is involved in amyloid production, formation of amyloid plaques and *APOE*/amyloid complexes, cellular clearance of amyloid, amyloid clearance through the blood-brain barrier, and proteolytic degradation [8, 9, 37]. Given the numerous mechanisms of e4 influencing amyloid, it is no surprise that amyloid PET studies consistently demonstrate that e4 is associated with an earlier onset of amyloid accumulation [38] as well as increased amyloid deposition rate and burden [6, 7, 39, 40]. Our results are consistent with these studies as we show that e4 dosage is associated with increased risk of amyloid positivity as well as continuous amyloid burden even after amyloid positivity is reached. Specifically, each e4 and e2 allele was associated with +15 and −12 centiloids, respectively, in Aβ+ CU. Given these effects, it is critical to account for continuous amyloid burden in analyses examining the impact of *APOE* on regional tau burden [41–44].

Our finding that the effects of e4 on amyloid burden are greater than the protective effects of e2 for amyloid positivity is consistent with mechanistic work that shows more pathways linking e4 to amyloid processing than e2 [9, 37]. For instance, whereas both e4 and e2 have been associated with amyloid plaques and amyloid clearance [45], e4 may additionally affect extracellular *APOE*/amyloid complexes [46], cellular clearance of amyloid [47, 48], and proteolytic degradation of monomeric amyloid [49]. Overall, our analyses of the full A4/LEARN screening dataset of 4486 CU confirmed the expected effects of e4 and e2 on both overall amyloid positivity and continuous amyloid burden within the Aβ+ CU group.

Our main finding was that both e4 and e2 influence early regional tau levels among Aβ+ individuals. Across two datasets (i.e., 392 Aβ+ CU from A4/LEARN; 220 Aβ+ CU and MCI from ADNI), we showed that e4 was associated with higher MTL tau PET burden, an effect that was only partially mediated by continuous amyloid burden. Interestingly, the overall pattern of significant direct effects of e4 on MTL accounting for ~50% of the total effect was consistent between Aβ+ CU data from A4/LEARN and combined Aβ+ CU and MCI data from ADNI. One difference between the A4/LEARN and ADNI results was that the overall magnitude of the total e4 effect on regional tau in MTL and neocortical regions was 2–3 times stronger in ADNI (increase of 0.039–0.152 SUVR per allele) than A4/LEARN (increase in 0.019–0.073 SUVR per allele). The stronger total effects in ADNI can be explained by the inclusion of Aβ+ MCI in the ADNI analyses, who are known to have a greater range of tau PET values than Aβ+ CU [27, 50]. Despite differing magnitudes, the fact that the proportion of e4 direct and indirect effects remained consistent in both cohorts provides further support for an association between e4 on tau during the pre-dementia stages of disease. Potential mechanisms may involve mislocalization of tau from the axon to the soma and dendrites, promotion of aberrant hyperphosphorylation, acceleration of spread from diseased to healthy neurons, enhancement of aggregation of p-tau into insoluble neurofibrillary tangles, and disruption of tau clearance due to e4 [9, 51].

In addition to e4 effects on regional tau, we also found that e2 was associated with reduced tau levels in both MTL and neocortical regions. More than 75% of the total effect of e2 on tau in MTL and cortex was direct (i.e., not accounted for by continuous amyloid burden). Our findings are consistent with animal models and iPSC-derived human brain cell cultures that have identified direct associations between *APOE* and tau accumulation [51–55]. *APOE*2 in particular may be protective against AD partially through lipid and metabolic mechanisms that lead to fewer tau tangles [44]. It is likely that other non-amyloid factors have a similar impact on disease progression, accelerating progression in some Aβ+ individuals but allowing for resilience to downstream changes in other Aβ+ individuals [56]. Given the link between elevated tau burden and risk of clinical progression from CU to MCI [14], the identification of factors that influence tau burden among Aβ+s will improve individual level prediction and shed insight into mechanisms of disease risk and resilience.

Our results also show that e4 and e2 differ in their impact on tau spatial patterns. Specifically, e4 was only linked to regional effects within the MTL whereas the effect of e2 was consistent across MTL and neocortical regions. Our findings linking e4 specifically to MTL tau is consistent with several studies combining participants across the AD clinical spectrum [16, 21–23]. Mattsson and colleagues [21] reported that in AD dementia patients, e4 carriers were similar to non-carriers in the magnitude of regional tau, but e4 carriers had greater entorhinal cortex uptake relative to neocortical uptake quantified using a ratio. *APOE*4 is known to lead to blood-brain barrier dysfunction [57–60] and regional susceptibility to blood-brain barrier breakdown [61] may explain the MTL dominant pattern of tau in e4 carriers. While blood-brain barrier dysfunction contributes to cognitive decline independent of AD pathology [62], there are also inflammatory and immune-related pathways linking e4 to Aβ and tau [63, 64]. Recent human work linking blood-brain barrier dysfunction to *APOE*4 status showed selective blood-brain barrier dysfunction in the MTL [62], suggesting that the MTL in particular may have increased susceptibility to e4-related blood-brain barrier breakdown in aging. Our results extend these findings to provide further support for a selective effect of e4 on the MTL and suggest that this effect emerges during the preclinical stage of AD.

In A4/LEARN, we found that e2 was associated with a global reduction in tau burden across the MTL as well as all three neocortical regions. Given this more global pattern, it may be fruitful for future studies to explore e2 associations with cerebrospinal fluid or plasma biomarkers. To our knowledge, this is the first study to show a global effect of e2 on regional tau levels in preclinical AD. In vivo PET imaging and postmortem findings have provided evidence for regional variation in tau pathology that was associated with factors aside from amyloid such as differing levels of *APOE* expression, Related Orphan Receptor B positive neurons, homeostatic astrocytes, and inflammatory microglia function [65]. Although our effects are predominantly independent of continuous amyloid burden as measured by PET, the e2 effect we observed may still be mediated by amyloid-related processes. Given that e2 has been associated with slower longitudinal amyloid accumulation over time [6], it is possible that slower rates of accumulation are also associated with less downstream tau accumulation. For instance, a slower rate of abnormal amyloid deposition may afford greater opportunities for compensatory mechanisms and/or a more effective response to neuronal injury.

A recent case report highlighted a PSEN1-E280A individual with two copies of the *Apoe* Christchurch mutation, low to intermediate levels of tau burden, extremely high levels of amyloid, and preserved cognition for nearly 30 years beyond the typical age of AD symptoms for the PSEN1-E280A kindred [65]. Although the *Apoe* Christchurch mutation is only a candidate protective variant in this case, this mutation is thought to act through similar mechanisms to e2. Both the *Apoe* Christchurch mutation and e2 homozygosity are associated with Type III hyperlipoproteinemia [66] and lipid metabolism could potentially be an APOE-related protective mechanisms against significant tau accumulation. Overall, e2 seems to exert additional protection against AD dementia risk via pathways directly related to tau accumulation, although future work is needed to identify exact underlying mechanisms.

Although we showed that both e4 and e2 influence regional tau levels, differences in the overall pattern of these findings highlight that underlying mechanistic pathways may differ across *APOE* genotypes. While e2, e3, and e4 show a stepwise association with risk of clinical AD dementia as well as overall amyloid plaque burden [33, 48, 67], the impact of e2 on Aβ-related and Aβ-independent mechanisms of AD are less established than e4 effects [37]. This lack of understanding may be because e2 is less common than e4 in the general population. The expected frequency of e2 and e4 is approximately 8 and 13%, respectively, in individuals with European ancestry [68, 69], but e2 is especially underrepresented among Aβ+ CU (4–8% [29–31]) and AD dementia patients (approximately 11% [70]). Importantly, the rarity of e2 carriers among Aβ+ individuals limits the ability to evaluate e2-related mechanisms that influence disease progression above and beyond amyloid abnormalities in humans, such as downstream tau accumulation. This was evident in our analyses with ADNI, where the presence of only 13 e2 Aβ+ individuals precluded analysis (however, see previous work in ADNI that found no effect between e2 and tau PET when examining e2 effects in a combined group of Aβ- and Aβ+ CU and MCI [32]). The large sample size of the A4/LEARN CU dataset provided the opportunity to evaluate 31 Aβ+ e2 carriers that also have tau PET (1 e2/e2, 17 e2/e3, 13 e2/e4), which is still a small sample but nevertheless is larger than previous work. Given limited sample sizes for each e2 genotype, our general approach was to model e2 allele count, and additionally confirm results by directly contrasting e2/e3 and e3/e3 groups in sensitivity analyses. Future work that combines data across cohorts may provide one strategy to validate our A4/LEARN e2 findings and further characterize the impact of e2 on non-amyloid pathways.

There are several limitations to consider. First, only cross-sectional amyloid and cross-sectional tau data were examined. Because *APOE* genotype has been shown to influence rates of amyloid accumulation over time [6], it is possible that differences in tau burden relate to amyloid rate rather than *APOE* genotype. Similarly, it has been suggested that tau pathology begins to accelerate, particularly in neocortex, at some point after an individual becomes amyloid positive [15, 71]. Because *APOE*4 and *APOE*2 carriers tend to become amyloid positive at earlier and later ages, respectively, compared to *APOE*3 carriers [2, 72], it is possible that e2 carriers have been amyloid positive for a shorter period of time and that reduced tau reflects this shorter period rather than a direct effect on tau. Longitudinal data and/or modeling of amyloid duration [73–76] will be helpful for understanding the contribution of amyloid rate and duration. Second, although this is the largest cohort of preclinical AD participants to date, the limited number of Aβ+ e2 carriers that underwent tau PET reduces the power and reliability of our findings. This may be especially true for examinations of hypothesized age and sex interactions, which requires further subgrouping within the sample of 31 Aβ+ CU e2 carriers. However, autopsy and cohort studies have shown that e2 carriers are less likely to ever be Aβ+. Third, amyloid PET is unable to distinguish between amyloid plaques relevant to AD and amyloid buildup in arteries indicative of cerebral amyloid angiopathy (CAA), and there are known *APOE* genotype differences in CAA [77, 78]. Thus, if amyloid PET is capturing CAA-relevant amyloid in the A4/LEARN and ADNI samples, our analyses may not accurately capture the magnitude of amyloid-mediated effects of *APOE* on tau. Relatedly, amyloid PET tracers bind to amyloid plaques whereas [18F]-flortaucipir binds to neuropil threads, ghost tangles, and neuritic plaques [79]. There may be additional links between *APOE* and other types of amyloid and tau deposits, such as oligomers, that are not captured by PET and thus not assessed in this study. Finally, because we only examined Aβ+ individuals, we are unable to determine whether direct *APOE* effects on tau only emerge after amyloid positivity has been reached or whether the same direct *APOE* effects on tau would be present in the absence of amyloid. Although elevated tau PET values are sparse among Aβ- individuals, these elevations have been associated with reduced cognitive performance and greater atrophy [80]. Thus, it is possible that *APOE* exerts effects even among Aβ- individuals, warranting future larger studies that can determine whether effects of *APOE* on tau pathology are dependent on the presence of abnormal amyloid.

## Conclusions

Our results suggest that *APOE* genotype influences regional tau PET burden in the early stages of AD pathological accumulation. E4 was specifically associated with elevations in the MTL, whereas protective e2 effects were present in the MTL and neocortex. Given our findings for both amyloid-mediated and direct effects of *APOE* on tau, gene therapies [81–83] that leverage protective e2 mechanisms may have a global protective impact on limiting amyloid and tau across the brain. Our results also suggest that targeting amyloid removal in e4 carriers at the preclinical stage may not be enough for impacting downstream tau accumulation given that amyloid burden only explained 22–39% of the total effect of e4 on regional tau in the MTL. *APOE* effects on tau accumulation in Aβ+ CU highlight that across individuals, there are variable rates of progression throughout the AD cascade, as opposed to a uniform canonical pathway that follows from amyloid positivity. Treatments may need to target different mechanisms depending on disease stage, and these treatments may vary as a function of *APOE* genotype. For example, whereas anti-amyloid treatments may be effective among e3 and e2 Aβ+ individuals, e4 carriers may only benefit from anti-amyloid treatments before amyloid positivity is reached (and may require combination therapies targeting both amyloid and tau after amyloid positivity). Understanding *APOE* mechanisms on tau may inform the development of anti-tau treatments and more broadly, therapeutic strategies targeting underlying mechanisms related to *APOE* may be effective in preventing amyloid-mediated and direct effects of *APOE* on tau accumulation.

## Supplementary Information

**Additional file 1: Supplementary Methods. Description of how amyloid SUVRs are converted to centiloids. Table S1.** Demographic information for the 392 Aβ+ CU individuals with tau PET imaging from A4/LEARN. **Table S2.** Association between amyloid burden (centiloids) and regional tau SUVRs in (A) Aβ- and Aβ+  participants and in (B) only Aβ+ participants from A4/LEARN. Unstandardized beta values (SE), *p*-values, and model R^2^ values are listed. Each column represents a separate regression model. **Table S3.***APOE* associations with regional tau SUVRs in 392 Aβ+s with APOE*Age and APOE*Sex interactions included in the model. Unstandardized beta values (SE) and p-values are listed. Each column represents a separate regression model. **Table S4.** (A) *APOE* associations with regional tau SUVRs in 359 Aβ+ individuals (B) with APOE*age and APOE*sex interactions after excluding *APOE* homozygotes (1 e2/e2 participant and 25 e4/e4 participants). Unstandardized beta values (SE) and p-values are listed. Each column represents a separate regression model. **Table S5.** (A) *APOE* associations with regional tau SUVRs in 372 Aβ+ individuals (B) with APOE*age and APOE*sex interactions after excluding 13 e2/e4 participants. Unstandardized beta values (SE) and p-values are listed. Each column represents a separate regression model. **Table S6.** Comparisons of regional tau SUVRs between *APOE* (A) e2/e3 and e3/e3 groups as well as (B) e3/e4 and e3/e3 groups after adjusting for age and sex. Unstandardized beta values (SE) and *p*-values are listed. Each column represents a separate regression model. **Figure S1.** Conversion of global SUVRs to centiloids (CL). **(A)** Conversion of florbetapir (FBP) global amyloid SUVRs to CLs for A4/LEARN data. **(B)** Conversion of FBP and florbetaben (FBB) global amyloid SUVRs to CLs for ADNI data. **(C)** CL distributions across A4/LEARN FBP, ADNI FBP, and ADNI FBB data. **Figure S2.** Mediation models examining direct and indirect effects of *APOE* on regional tau SUVRs with continuous amyloid (centiloids) as a mediator in (A) Aβ+ clinically unimpaired (CU) individuals from A4 and (B) Aβ+ CU and mild cognitive impairment (MCI) individuals from ADNI. Both e4 and e2 effects were examined in A4, but only e4 effects were examined in ADNI due to the small sample size of e2 Aβ+ participants in ADNI (*n* = 13). Unstandardized betas are listed. Note: * *p* < 0.05, ** *p* < 0.01, *** *p* < 0.001. **Figure S3.** Mediation models examining the effects of *APOE*4 on regional tau SUVRs with continuous amyloid (centiloids) as a mediator among 162 Aβ+ CU and mild cognitive impairment (MCI) participants who had tau and amyloid PET scans within 2 years of each other. Results are consistent with the effects in the larger ADNI sample. Note: * *p* < 0.05, ** *p* < 0.01, *** *p* < 0.001.

## Data Availability

The datasets generated and/or analyzed during the current study are available through LONI as well as request to the corresponding author.

## Abbreviations

A4: Anti-Amyloid Treatment in Asymptomatic AD
AD: Alzheimer’s disease
*APOE*: Apolipoprotein
ADNI: Alzheimer’s Disease Neuroimaging Initiative
CAA: Cerebral amyloid angiopathy
CL: Centiloid
CU: Clinically unimpaired
FBB: Florbetaben
FBP: Florbetapir
LEARN: Longitudinal Evaluation of Amyloid Risk and Neurodegeneration
MCI: Mild cognitive impairment
MTL: Medial temporal lobe
PET: Positron emission tomography
SUVR: Standardized uptake value ratio
