# Supplementary material for: APOE effects on regional tau in preclinical Alzheimer’s disease
Source: Mol Neurodegener. 2023 Jan 4;18:1. doi: 10.1186/s13024-022-00590-4 (PMC9811772; doi:10.1186/s13024-022-00590-4)
Supplement: Supplementary file 1 — Additional file 1: Supplementary Methods. Description of how amyloid SUVRs are converted to centiloids. Table S1. Demographic information for the 392 Aβ+ CU individuals with tau PET imaging from A4/LEARN. Table S2. Association between amyloid burden (centiloids) and regional tau SUVRs in (A) Aβ- and Aβ+ participants and in (B) only Aβ+ participants from A4/LEARN. Unstandardized beta values (SE), p-values, and model R2 values are listed. Each column represents a separate regression model. Table S3.APOE associations with regional tau SUVRs in 392 Aβ+s with APOE*Age and APOE*Sex interactions included in the model. Unstandardized beta values (SE) and p-values are listed. Each column represents a separate regression model. Table S4. (A) APOE associations with regional tau SUVRs in 359 Aβ+ individuals (B) with APOE*age and APOE*sex interactions after excluding APOE homozygotes (1 e2/e2 participant and 25 e4/e4 participants). Unstandardized beta values (SE) and p-values are listed. Each column represents a separate regression model. Table S5. (A) APOE associations with regional tau SUVRs in 372 Aβ+ individuals (B) with APOE*age and APOE*sex interactions after excluding 13 e2/e4 participants. Unstandardized beta values (SE) and p-values are listed. Each column represents a separate regression model. Table S6. Comparisons of regional tau SUVRs between APOE (A) e2/e3 and e3/e3 groups as well as (B) e3/e4 and e3/e3 groups after adjusting for age and sex. Unstandardized beta values (SE) and p-values are listed. Each column represents a separate regression model. Figure S1. Conversion of global SUVRs to centiloids (CL). (A) Conversion of florbetapir (FBP) global amyloid SUVRs to CLs for A4/LEARN data. (B) Conversion of FBP and florbetaben (FBB) global amyloid SUVRs to CLs for ADNI data. (C) CL distributions across A4/LEARN FBP, ADNI FBP, and ADNI FBB data. Figure S2. Mediation models examining direct and indirect effects of APOE on regional tau SUVRs with continuous amyloid (cen [file 13024_2022_590_MOESM1_ESM.docx]

**Supplementary Methods**

*Converting amyloid SUVRs to centiloids (CLs):*

A4/LEARN and ADNI global amyloid PET SUVRs were converted to CLs using equations published in Royse et al. 2021 [1]. These conversion equations enabled amyloid PET SUVRs extracted from data processed with the ADNI FreeSurfer v7 MRI-based pipeline to be converted to a common PiB scale that has been processed with a “standard pipeline” described by Klunk et al. 2015 [2]. In other words, these equations enable global amyloid SUVRs obtained from different processing pipelines and different ligands to be placed onto a common scale. Although A4/LEARN amyloid data were processed locally at Stanford using a pipeline that mimicked the ADNI FreeSurfer v7 MRI-based pipeline [3], it is possible that slight differences in implementation in comparison to Royse et al. 2021 [1] may result in differing SUVR values. Thus, we first performed an internal validation step where FBP data from the Rowe et al. 2017 [4] FBP-PiB paired dataset were downloaded from the Global Alzheimer’s Association Interactive Network (GAAIN) and processed using our local pipeline [3]. We then followed identical procedures described by Royse et al. 2021 [1], regressing our locally processed FBB SUVRs against the PiB standard SUVRs calculated by Rowe et al. 2017 [4]. This step yielded a near identical regression fit to Royse et al. 2021 [1] (i.e., our regression equation: y = 0.499x + 0.503, R^2^ = 0.880 vs. the Royse et al. 2021 regression equation in Figure 2: y = 0.497x + 0.503, R^2^ = 0.897). We therefore applied the Royse et al. 2021 [1] equation to our locally processed A4/LEARN amyloid PET data. For the ADNI data, since FBP and FBB values from the ADNI FreeSurfer v7 MRI-based pipeline were directly downloaded from LONI, the processing pipeline is identical to what is described in Royse et al. 2021 [1], ensuring that the equations can easily be applied to the ADNI SUVRs. With these procedures, FBP data collected from A4/LEARN as well as FBP and FBB data collected from ADNI were placed onto the same comparable scale (**Figure S1**).

**Table S1.** Demographic information for the 392 Aβ+ CU individuals with tau PET imaging from A4/LEARN.

|  | *APOE* Genotype | | | | | | |
| --- | --- | --- | --- | --- | --- | --- | --- |
|  | e2/e2 (n=1) | e2/e3 (n=17) | e2/e4 (n=13) | e3/e3 (n=147) | e3/e4 (n=182) | e4/e4 (n=25) | Missing (n=7) |
| Age, mean (SD) | 68.7 (NA) | 71.7 (4.9) | 71.9 (6.2) | 73.0 (5.0) | 71.8 (4.7) | 69.7 (3.4) | 72.1 (4.8) |
| Sex, n (%) |  |  |  |  |  |  |  |
| Male | 0 (0%) | 9 (53%) | 7 (54%) | 64 (44%) | 76 (42%) | 6 (24%) | 167 (43%) |
| Female | 1 (100%) | 8 (47%) | 6 (46%) | 83 (57%) | 106 (58%) | 19 (76%) | 225 (57%) |
| Amyloid Centiloid, mean (SD) | 42.1 (NA) | 34.1 (19.6) | 43.4 (29.2) | 46.2 (28.5) | 62.5 (28.6) | 66.9 (37.6) | 65.3 (32.0) |

**Table S2.** Association between amyloid burden (centiloids) and regional tau SUVRs in (A) Aβ- and Aβ+ participants and in (B) only Aβ+ participants from A4/LEARN. Unstandardized beta values (SE), *p*-values, and model R^2^ values are listed. Each column represents a separate regression model.

|  | Entorhinal | Amygdala | Inferior Temporal | Inferior Parietal | Precuneus |
| --- | --- | --- | --- | --- | --- |
| (A) All participants (55 Aβ- and 392 Aβ+) | | | | | |
| Age | 0.000 (0.001) p=0.979 | 0.002 (0.002) p=0.279 | 0.000 (0.001) p=0.926 | **-0.003 (0.001) p=0.003** | -0.001 (0.001) p=0.154 |
| Sex  (F vs. M) | 0.019 (0.014) p=0.184 | -0.004 (0.015) p=0.771 | 0.010 (0.011) p=0.380 | **0.036 (0.011) p<0.001** | 0.006 (0.009) p=0.526 |
| Amyloid burden | **0.002 (0.000) p<0.001** | **0.002 (0.000) p<0.001** | **0.002 (0.000) p<0.001** | **0.001 (0.000) p<0.001** | **0.001 (0.000) p<0.001** |
| Model R^2^ | 0.196 | 0.145 | 0.164 | 0.167 | 0.094 |
| (B) 392 Aβ+ only | | | | | |
| Age | -0.000 (0.002) p=0.947 | 0.002 (0.002) p=0.239 | -0.000 (0.001) p=0.870 | **-0.004 (0.001) p=0.001** | -0.001 (0.001) p=0.225 |
| Sex  (F vs. M) | 0.027 (0.016) p=0.094 | -0.002 (0.016) p=0.880 | 0.012 (0.013) p=0.358 | **0.038 (0.012) p=0.002** | 0.003 (0.010) p=0.765 |
| Amyloid burden | **0.002 (0.000) p<0.001** | **0.002 (0.000) p<0.001** | **0.002 (0.000) p<0.001** | **0.001 (0.000) p<0.001** | **0.001 (0.000) p<0.001** |
| Model R^2^ | 0.144 | 0.108 | 0.136 | 0.145 | 0.068 |

**Table S3**. *APOE* associations with regional tau SUVRs in 392 Aβ+s with *APOE**Age and *APOE**Sex interactions included in the model. Unstandardized beta values (SE) and p-values are listed. Each column represents a separate regression model.

|  | Entorhinal | Amygdala | Inferior Temporal | Inferior Parietal | Precuneus |
| --- | --- | --- | --- | --- | --- |
| *APOE*2 | -0.080 (0.042) p=0.057 | -0.080 (0.042) p=0.055 | **-0.078 (0.034) p=0.024** | -0.045 (0.032) p=0.164 | **-0.074 (0.025) p=0.004** |
| *APOE*4 | **0.055 (0.022) p=0.014** | **0.063 (0.022) p=0.004** | 0.033 (0.018) p=0.067 | **0.048 (0.017) p=0.005** | 0.023 (0.013) p=0.080 |
| Age | **0.005 (0.002) p=0.025** | **0.007 (0.002) p=0.003** | 0.003 (0.002) p=0.112 | 0.001 (0.002) p=0.738 | 0.003 (0.001) p=0.057 |
| Sex (F vs. M) | 0.029 (0.024) p=0.242 | -0.012 (0.024) p=0.627 | 0.011 (0.020) p=0.575 | **0.053 (0.019) p=0.005** | 0.003 (0.015) p=0.829 |
| *APOE*2 * Age | -0.007 (0.006) p=0.194 | **-0.011 (0.006) p=0.045** | -0.001 (0.005) p=0.812 | -0.005 (0.004) p=0.304 | **-0.008 (0.003) p=0.014** |
| *APOE*4 * Age | -0.002 (0.003) p=0.493 | -0.001 (0.003)  p=0.831 | -0.000 (0.002) p=0.837 | -0.003 (0.002) p=0.223 | -0.003 (0.002) p=0.125 |
| *APOE*2*Sex (F vs. M) | -0.010 (0.058) p=0.866 | -0.002 (0.058) p=0.974 | 0.044 (0.047) p=0.350 | 0.004 (0.045) p=0.927 | 0.039 (0.035)  p=0.263 |
| *APOE*4 * Sex (F vs. M) | 0.005 (0.028) p=0.861 | 0.019 (0.028) p=0.500 | 0.002 (0.023) p=0.915 | -0.022 (0.022) p=0.307 | -0.004 (0.017) p=0.829 |

**Table S4.** (A) *APOE* associations with regional tau SUVRs in 359 Aβ+ individuals (B) with *APOE**age and *APOE**sex interactions after excluding *APOE* homozygotes (1 e2/e2 participant and 25 e4/e4 participants). Unstandardized beta values (SE) and p-values are listed. Each column represents a separate regression model.

|  | Entorhinal | Amygdala | Inferior Temporal | Inferior Parietal | Precuneus |
| --- | --- | --- | --- | --- | --- |
| (A) Predicting regional tau | | | | | |
| *APOE*2 | **-0.092 (0.029) p=0.002** | **-0.092 (0.028) p=0.001** | **-0.057 (0.023) p=0.016** | **-0.048 (0.022) p=0.031** | **-0.062 (0.017) p<0.001** |
| *APOE*4 | **0.057 (0.016) p<0.001** | **0.075 (0.016) p<0.001** | **0.032 (0.013) p=0.014** | **0.029 (0.012) p=0.020** | **0.023 (0.010) p=0.016** |
| Age | **0.004 (0.002) p=0.021** | **0.006 (0.002) p<0.001** | **0.003 (0.001) p=0.015** | -0.001 (0.001) p=0.618 | 0.001 (0.001) p=0.410 |
| Sex (F vs. M) | 0.029 (0.017) p=0.083 | -0.002 (0.016) p=0.892 | 0.017 (0.013) p=0.204 | **0.040 (0.013) p=0.001** | 0.003 (0.010) p=0.796 |
| (B) Predicting regional tau with *APOE**Age and *APOE**Sex Interactions | | | | | |
| *APOE*2 | -0.081 (0.041) p=0.051 | **-0.078 (0.040) p=0.048** | **-0.076 (0.033) p=0.023** | -0.045 (0.031) p=0.151 | **-0.075 (0.024) p=0.002** |
| *APOE*4 | **0.063 (0.025) p=0.014** | **0.060 (0.024) p=0.014** | 0.026 (0.020) p=0.201 | **0.050 (0.019) p=0.009** | **0.038 (0.015) p=0.010** |
| Age | **0.005 (0.002) p=0.043** | 0.006 (0.002) p=0.019 | 0.002 (0.002) p=0.295 | -0.001 (0.002) p=0.768 | 0.002 (0.001) p=0.092 |
| Sex (F vs. M) | 0.034 (0.025) p=0.173 | -0.010 (0.024) p=0.680 | 0.012 (0.020) p=0.549 | **0.061 (0.019) p=0.001** | 0.010 (0.014) p=0.472 |
| *APOE*2 * Age | -0.008 (0.006) p=0.186 | **-0.012 (0.005) p=0.024** | -0.002 (0.005) p=0.673 | -0.005 (0.004) p=0.242 | **-0.009 (0.003) p=0.009** |
| *APOE*4 * Age | -0.001 (0.003) p=0.829 | 0.004 (0.003) p=0.196 | 0.003 (0.003) p=0.297 | 0.000 (0.003) p=0.867 | -0.002 (0.002) p=0.407 |
| *APOE*2*Sex (F vs. M) | -0.013 (0.060) p=0.821 | -0.016 (0.057) p=0.783 | 0.040 (0.048) p=0.399 | -0.001 (0.045) p=0.983 | 0.038 (0.034) p=0.275 |
| *APOE*4 * Sex (F vs. M) | -0.008 (0.033) p=0.813 | 0.021 (0.032) p=0.506 | 0.006 (0.026) p=0.834 | -0.038 (0.025) p=0.128 | -0.022 (0.019) p=0.256 |

**Table S5.** A) *APOE* associations with regional tau SUVRs in 372 Aβ+ individuals (B) with *APOE**age and *APOE**sex interactions after excluding 13 e2/e4 participants. Unstandardized beta values (SE) and *p*-values are listed. Each column represents a separate regression model.

|  | Entorhinal | Amygdala | Inferior Temporal | Inferior Parietal | Precuneus |
| --- | --- | --- | --- | --- | --- |
| (A) Predicting regional tau | | | | | |
| *APOE*2 | **-0.073 (0.036) p=0.041** | **-0.079 (0.036) p=0.029** | **-0.054 (0.029) p=0.068** | -0.033 (0.028) p=0.238 | **-0.046 (0.022) p=0.036** |
| *APOE*4 | **0.059 (0.014) p<0.001** | **0.074 (0.014) p<0.001** | **0.034 (0.011) p=0.003** | **0.035 (0.011) p=0.001** | **0.021 (0.008) p=0.015** |
| Age | **0.004 (0.002) p=0.018** | **0.006 (0.002) p<0.001** | 0.003 (0.001) p=0.065 | -0.001 (0.001) p=0.433 | 0.001 (0.001) p=0.415 |
| Sex (F vs. M) | **0.033 (0.017) p=0.048** | -0.001 (0.017) p=0.941 | 0.012 (0.014) p=0.364 | **0.040 (0.013) p=0.002** | 0.004 (0.010) p=0.665 |
| (B) Predicting regional tau with *APOE**Age and *APOE**Sex Interactions | | | | | |
| *APOE*2 | -0.075 (0.057) p=0.193 | -0.087 (0.058) p=0.131 | -0.041 (0.047) p=0.381 | -0.005 (0.044) p=0.912 | -0.059 (0.035) p=0.092 |
| *APOE*4 | **0.056 (0.023) p=0.016** | **0.062 (0.023) p=0.008** | **0.039 (0.019) p=0.042** | **0.054 (0.018) p=0.003** | 0.026 (0.014) p=0.068 |
| Age | **0.005 (0.002) p=0.031** | 0.007 (0.002) p=0.004 | 0.003 (0.002) p=0.090 | 0.001 (0.002) p=0.673 | 0.003 (0.002) p=0.042 |
| Sex (F vs. M) | 0.028 (0.025) p=0.255 | -0.013 (0.025) p=0.595 | 0.016 (0.020) p=0.433 | **0.057 (0.019) p=0.003** | 0.004 (0.015) p=0.772 |
| *APOE*2 * Age | -0.005 (0.009) p=0.542 | -0.013 (0.009) p=0.137 | -0.006 (0.007) p=0.367 | -0.008 (0.007) p=0.258 | **-0.010 (0.005) p=0.047** |
| *APOE*4 * Age | -0.002 (0.003) p=0.560 | -0.001 (0.003) p=0.782 | -0.001 (0.003) p=0.680 | -0.003 (0.002) p=0.195 | -0.003 (0.002) p=0.112 |
| *APOE*2*Sex (F vs. M) | 0.003 (0.077) p=0.972 | 0.011 (0.078) p=0.889 | -0.021 (0.063) p=0.736 | -0.045 (0.060) p=0.449 | 0.023 (0.047) p=0.625 |
| *APOE*4 * Sex (F vs. M) | -0.005 (0.029) p=0.849 | 0.021 (0.029) p=0.470 | -0.006 (0.024) p=0.807 | -0.029 (0.022) p=0.193 | -0.006 (0.018) p=0.743 |

**Table S6.** Comparisons of regional tau SUVRs between *APOE* (A) e2/e3 and e3/e3 groups as well as (B) e3/e4 and e3/e3 groups after adjusting for age and sex. Unstandardized beta values (SE) and *p*-values are listed. Each column represents a separate regression model.

|  | Entorhinal | Amygdala | Inferior Temporal | Inferior Parietal | Precuneus |
| --- | --- | --- | --- | --- | --- |
| (A) 17 e2/e3 and 147 e3/e3 carriers only | | | | | |
| e2/e3 vs. e3/e3 | **-0.075 (0.036) p=0.038** | **-0.094 (0.032) p=0.004** | **-0.060 (0.029) p=0.037** | -0.035 (0.027) p=0.196 | **-0.051 (0.020) p=0.011** |
| Age | **0.004 (0.002) p=0.046** | **0.004 (0.002) p=0.025** | 0.002 (0.002) p=0.307 | -0.001 (0.002) p=0.574 | 0.002 (0.001) p=0.193 |
| Sex (F vs. M) | 0.034 (0.022) p=0.116 | -0.009 (0.020) p=0.647 | 0.016 (0.018) p=0.353 | **0.062 (0.017) p<0.001** | 0.016 (0.012) p=0.182 |
| (B) 182 e3/e4 and 147 e3/e3 carriers only | | | | | |
| e3/e4 vs. e3/e3 | **0.061 (0.017) p<0.001** | **0.077 (0.017)**  **p<0.001** | **0.032 (0.014) p=0.019** | **0.031  (0.013) p=0.017** | **0.026 (0.010) p=0.011** |
| Age | **0.005 (0.002) p=0.009** | **0.008 (0.002) p<0.001** | **0.003 (0.001) p=0.015** | -0.000 (0.001) p=0.957 | 0.002 (0.001)  p=0.102 |
| Sex (F vs. M) | 0.030 (0.017) p=0.085 | -0.001 (0.017) p=0.958 | 0.013 (0.014) p=0.340 | **0.040 (0.013) p=0.003** | -0.001 (0.010) p=0.959 |

**Figure S1.** Conversion of global SUVRs to centiloids (CL). (**A**) Conversion of florbetapir (FBP) global amyloid SUVRs to CLs for A4/LEARN data. (**B**) Conversion of FBP and florbetaben (FBB) global amyloid SUVRs to CLs for ADNI data. (**C**) CL distributions across A4/LEARN FBP, ADNI FBP, and ADNI FBB data.


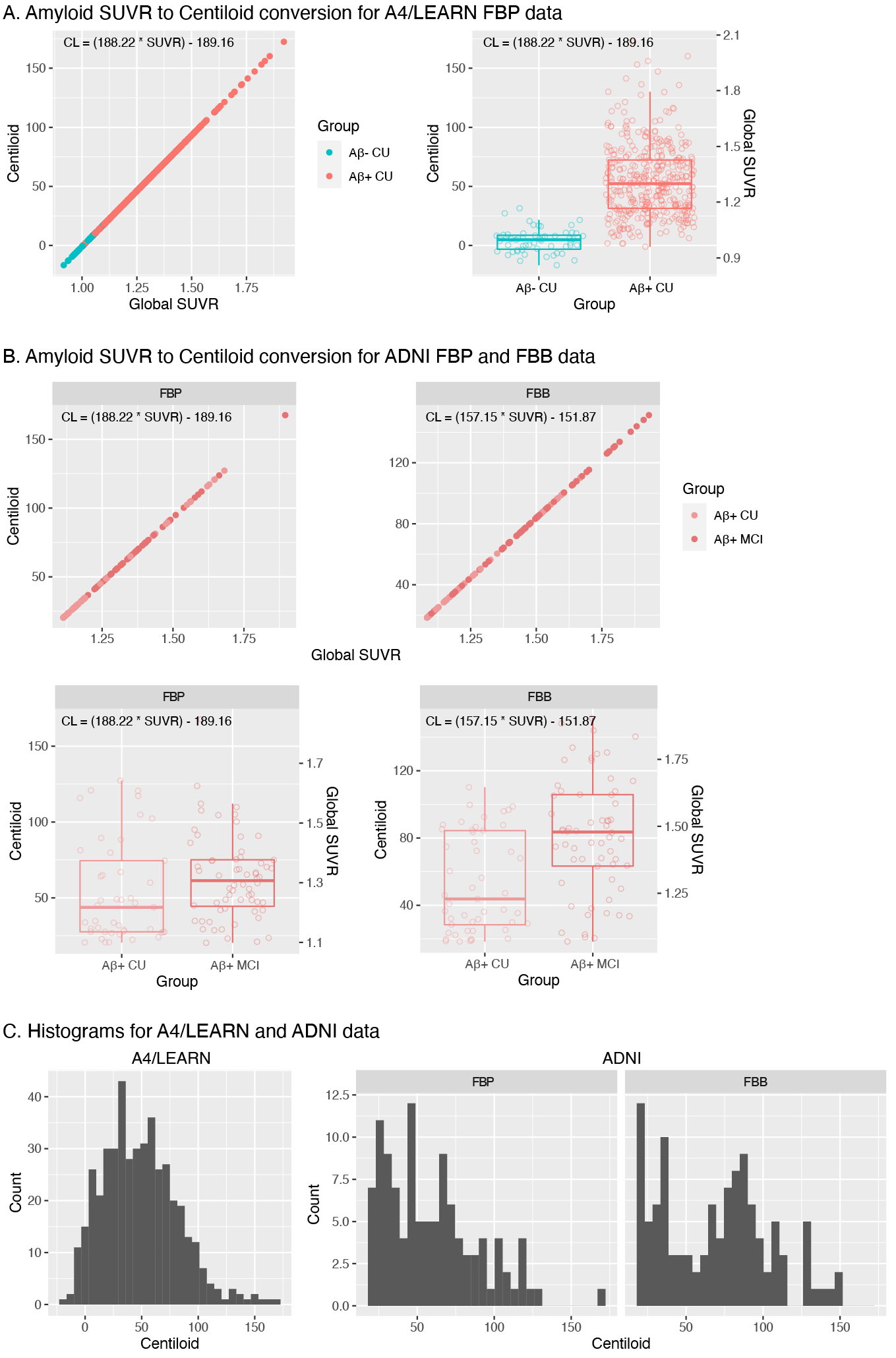


**Figure S2.** Mediation models examining direct and indirect effects of *APOE* on regional tau SUVRs with continuous amyloid (centiloids) as a mediator in (A) Aβ+ clinically unimpaired (CU) individuals from A4 and (B) Aβ+ CU and mild cognitive impairment (MCI) individuals from ADNI. Both e4 and e2 effects were examined in A4, but only e4 effects were examined in ADNI due to the small sample size of e2 Aβ+ participants in ADNI (*n* = 13). Unstandardized betas are listed. Note: * *p* < 0.05, ** *p* < 0.01, *** *p* < 0.001.


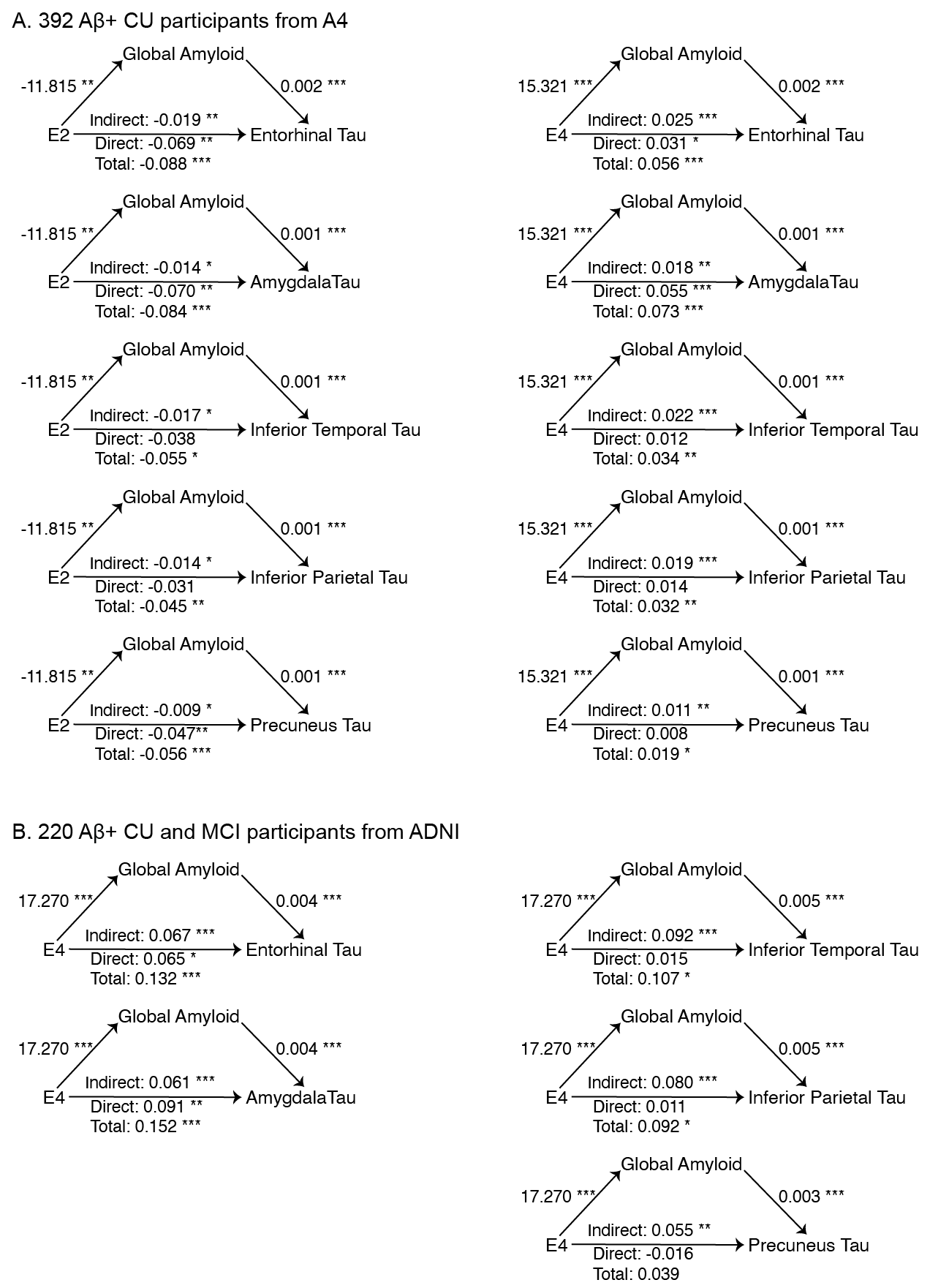


**Figure S3.** Mediation models examining the effects of *APOE*4 on regional tau SUVRs with continuous amyloid (centiloids) as a mediator among 162 Aβ+ CU and mild cognitive impairment (MCI) participants who had tau and amyloid PET scans within 2 years of each other. Results are consistent with the effects in the larger ADNI sample. Note: * *p* < 0.05, ** *p* < 0.01, *** *p* < 0.001.


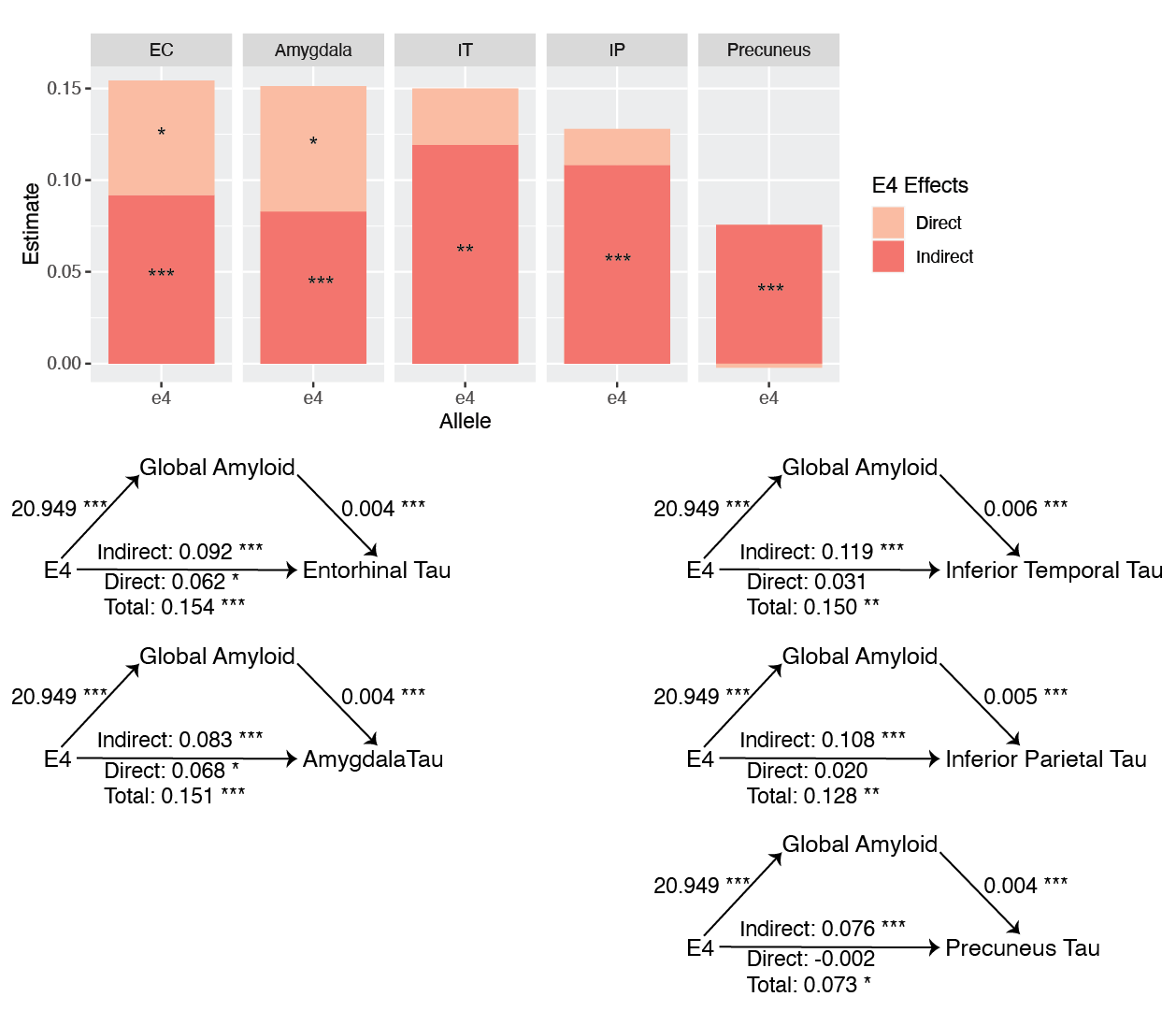


**References**

1. Royse SK, Minhas DS, Lopresti BJ, Murphy A, Ward T, Koeppe RA, et al. Validation of amyloid PET positivity thresholds in centiloids: a multisite PET study approach. Alzheimer’s Research & Therapy. 2021;13:99.

2. Klunk WE, Koeppe RA, Price JC, Benzinger TL, Devous MD, Jagust WJ, et al. The Centiloid Project: standardizing quantitative amyloid plaque estimation by PET. Alzheimers Dement. 2015;11:1-15.e1-4.

3. Young CB, Winer JR, Younes K, Cody KA, Betthauser TJ, Johnson SC, et al. Divergent Cortical Tau Positron Emission Tomography Patterns Among Patients With Preclinical Alzheimer Disease. JAMA Neurol. 2022;

4. Rowe CC, Doré V, Jones G, Baxendale D, Mulligan RS, Bullich S, et al. 18F-Florbetaben PET beta-amyloid binding expressed in Centiloids. Eur J Nucl Med Mol Imaging. 2017;44:2053–9.
